# A Review of Wet Compounding of Cellulose Nanocomposites

**DOI:** 10.3390/polym13060911

**Published:** 2021-03-16

**Authors:** Craig Clemons, Ronald Sabo

**Affiliations:** USDA Forest Products Laboratory, One Gifford Pinchot Drive, Madison, WI 53726, USA; ronald.sabo@usda.gov

**Keywords:** wet compounding, water-assisted compounding, wet feeding, cellulose nanomaterials

## Abstract

Cellulose nanomaterials (CNs) are an emerging class of materials with numerous potential applications, including as additives or reinforcements for thermoplastics. Unfortunately, the preparation of CNs typically results in dilute, aqueous suspensions, and the lack of efficient water removal methods has hindered commercialization. However, water may also present opportunities for improving overall efficiencies if its potential is better understood and if it is better managed through the various stages of CN and composite production. Wet compounding represents one such possible opportunity by leveraging water’s ability to aid in CN dispersion, act as a transport medium for metering and feeding of CNs, plasticize some polymers, or potentially facilitate the preparation of CNs during compounding. However, there are also considerable challenges and much investigation remains. Here, we review various wet compounding approaches used in the preparation of cellulose nanocomposites as well as the related concepts of wet feeding and wet extrusion fibrillation of cellulose. We also discuss potential opportunities, remaining challenges, and research and development needs with the ultimate goal of developing a more integrated approach to cellulose nanocomposite preparation and a more sophisticated understanding of water’s role in the compounding process.

## 1. Introduction

**Figure 1:**
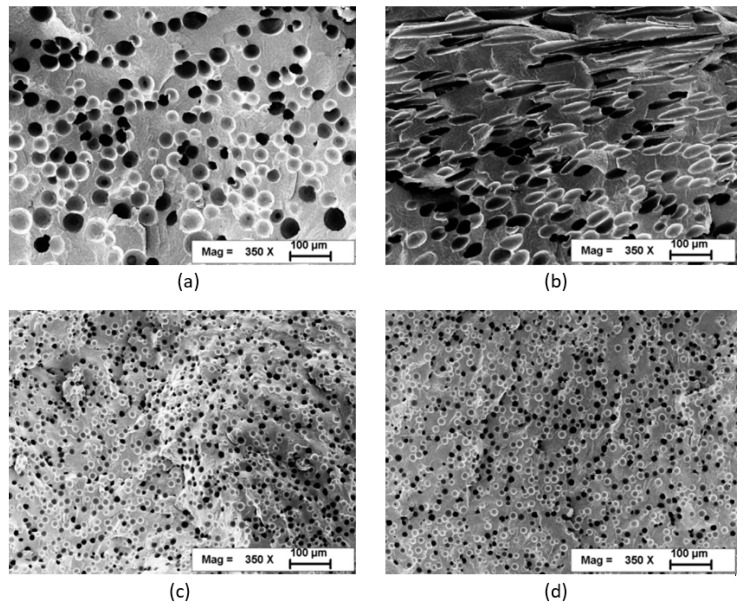
Scanning electron microscope images polyamide 6 with 0 (**a**,**b**) and 2% nanocellulose (**c**,**d**) fractured perpendicular (**a**,**c**) and parallel (**b**,**d**) to the melt flow direction. Reproduced with permission from, Peng, et al. [6], Polymer, Elsevier Ltd., 2016.

Cellulose nanomaterials (CNs) have received considerable recent attention as an emerging class of materials. Many potential applications are being investigated, including as reinforcements or additives such as foam nucleating agents (Figure 1) in thermoplastics. However, remaining technical and practical challenges hinder the ability to produce well-dispersed cellulose nanocomposites in a scalable manner. For example, the preparation of CNs typically results in dilute, aqueous suspensions, and the efficient removal of water from these suspensions represents a key obstacle to commercialization [1]. However, because of their strong propensity to hydrogen bond, complete drying at elevated or even room temperature can lead to irreversible agglomeration, yielding micron-scale or larger particles that may negate the benefit of using CNs in the first place. In the laboratory, dispersion/agglomeration challenges are often overcome by solvent exchanging, severe sonication, complex chemical modification, etc., [2,3,4,5]. However, these can be expensive, not particularly environmentally friendly, or not practical on a large scale. Freeze- or spray-drying has also been used to minimize hydrogen bonding but can be expensive and lead to low bulk density CNs that are difficult to feed and meter and that can still be difficult to thoroughly disperse in polymers.

Rather than simply being a technical hurdle and financial liability, water may be part of the solution or present opportunities if its potential can be better understood and if it can be better managed during the various stages of CN and composite production. For example, a growing body of recent research has demonstrated improved dispersion in CNs by wet compounding, some with only small quantities of water [6,7,8,9,10,11,12]. Wet (a.k.a. water-assisted) compounding has been previously explored for other types of nanocomposites, and there is much that could be learned and potentially applied to cellulose nanocomposites. Given that the preparation of CNs results in aqueous suspensions, there is additional motivation for such an approach, offering an opportunity to simultaneously dry and compound cellulose nanomaterials with polymers without the need for a standalone drying process. Such a holistic or integrated approach to CN and nanocomposite preparation could potentially offer processing efficiencies and reduce cost. 

The presence of water during compounding can also have other advantages besides aiding dispersion. For example, water can act as a transport medium for metering and feeding of CNs into compounding equipment (“wet feeding”), avoiding the challenges of the feeding and metering of a low bulk density powder. Such approaches are already of commercial interest for the safe handling of other nanomaterials such as carbon nanotubes [13]. Water can also act as plasticizer in hydrogen-bonded polymers such as polyamides (PAs), depressing the melting point of polyamide 6 (PA6) by as much as 60 °C (the “cryoscopic effect”), for example [14,15]. Such reductions can allow compounding of CNs with higher melting temperature polymers than would otherwise be possible due to CN’s limited thermal stability [8]. Water can also potentially perform other functions such as acting as a benign solvent or a reactant during reactive extrusion. Finally, there is also the potential for not only dispersing micro/nano-scale materials during a wet compounding process but also preparing them as well. In this case, water can act as a swelling agent and, in combination with an appropriate pretreatment, allow for the breakdown of the cell wall if sufficient energy is applied. Such a process would more likely lead to a highly microfibrillated cellulose rather than a true CN, but never-the-less may have useful properties. 

However, wet compounding is not without its challenges as well. The melting temperature of most common thermoplastics are well above the boiling point of water. If not handled appropriately, the removal of water can simply result in the formation of hydrogen-bonded cellulose agglomerates. Additionally, the presence of water has the potential to hydrolyze susceptible polymer matrices, additives, or treated CNs. Additional equipment may be needed such as specialized pumps to feed aqueous CN suspensions into high pressure zones of extruders or extruders may need to be extended to accommodate additional operations (e.g., devolatilization). Depending upon how much water removal is necessary, output rates may have to be reduced, increasing the costs. Also, wet compounding does not change the basic challenge of compounding a strongly hydrophilic nanomaterial to polymers, many of which are hydrophobic. To overcome this, CN treatments, additives, etc., are often still required and these need to be efficient, low cost, and compatible with water-based processing. Despite these challenges, recent progress has been made in using wet compounding for preparing cellulose nanocomposites and the approach appears promising. 

Below, we review CNs as well as what has been learned from wet compounding of nanoclays and its relevance to compounding of cellulose nanocomposites. We also review progress on wet compounding of cellulose nanocomposites thus far and identify potential opportunities and challenges, as well as technology gaps and research needs.

## 2. Discussion

### 2.1. Cellulose Nanomaterials

Cellulose nanomaterials are a relatively new class of bio-derived cellulosic materials consisting of nanoscale dimensions. These materials are of considerable and rapidly growing interest because they have numerous advantageous and/or unique properties, such as transparency, vapor barrier resistance, and high strength. Detailed descriptions of CNs can be found in recent reviews [3,4,16,17,18,19,20]. However, a brief introduction and discussion of CNs as they pertain to wet compounding is provided below. 

**Figure 2:**
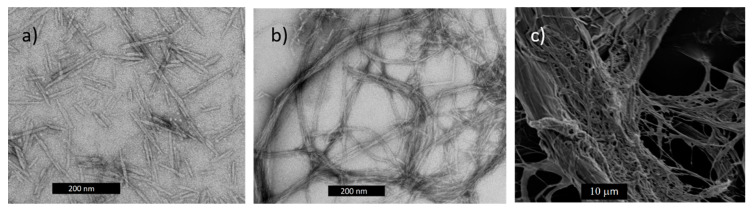
Various types of nano- and micro-cellulose: (**a**) cellulose nanocrystals (CNCs, scale bar = 200 nm); (**b**) cellulose nanofibrils (CNFs, scale bar = 200 nm); (**c**) microfibrillated cellulose (MFC, scale bar = 10 µm).

CNs have a wide range of morphologies (Figure 2), surface chemistries and properties but can generally be classified as either cellulose nanocrystals (CNCs) or cellulose nanofibrils (CNFs) [3,4,21]. CNCs are highly crystalline, discrete rod-shaped particles of nano-scale dimensions. They are typically produced by acid hydrolysis, which removes most of the amorphous cellulose and leaves behind the recalcitrant, largely crystalline portion of the original cellulose material. Though somewhat limited by their modest aspect ratio, CNCs are often touted for characteristics such as high strength, high stiffness, optical activity, and self-assembly, making them candidates for a large range of applications, including enhancing composites (Figure 2a). 

Conversely, CNFs consist more of networked structures of fibers than of discrete particles, and their applications range from rheological modifiers to substrates for flexible electronics to composite reinforcement. CNFs can be created by many methods but typically include some combination of chemical or biological treatment and mechanical refining. It is important to note that the morphology of fibrillated cellulose can vary from uniform, high-aspect ratio nanoscale fibers to coarse fibers that have extensive surface nano-scale fibrillation, depending on the process used to create them. For example, CNFs produced primarily from mechanical refining are networked, hierarchical structures with broad particle size distributions that have low viscosities and are mostly opaque (Figure 2c). These mechanical grade CNFs typically consist of fibers that are not completely nanoscale, but they are likely to be lower cost to produce than other types. Conversely, certain chemical pretreatments can lead to much finer fibrillation and highly transparent grades (Figure 2b). The terminology used for fibrillated celluloses is not always precise and such distinctions can be difficult to make because of the material’s hierarchical nature. Microfibrillated cellulose (MFC) is often used synonymously for CNFs and little distinction in terminology is made between materials consisting mostly of nanoscale fibers and those consisting of micron-scale fibers with some nano-scale fibers emanating from their surfaces. In this review, we include investigations that may not have strictly produced nanocomposites but that have relevance to producing cellulose nanocomposites by wet compounding. We use the term “CNF” for all such fibrillated celluloses as a matter of convenience.

Both CNCs and CNFs are predominantly produced as dilute aqueous suspensions that can be readily concentrated to about 1–10%, depending on the type of CN and method of preparation [22]. At this point, the suspensions exhibit yield stresses and shear-thinning behavior [23,24,25,26,27,28,29] and their high viscosities make further concentration by typical wet processing methods problematic. Concentrating CN suspensions further could mitigate some of the costs and challenges associated with handling dilute suspensions, including in composite preparation. Some grades are now offered at about 15% solids [22] and further concentrating (dewatering) of these suspensions is an on-going area of research [30].

### 2.2. Wet Compounding of Clay Nanocomposites

Prior to the recent growth of CNs, wet compounding approaches were applied to other types of nanomaterials such as nanoclays, and it is useful to consider what was learned and whether these approaches are applicable to cellulose nanocomposites. Here we limit our discussion to general concepts and certain aspects of relevance to cellulose nanocomposites. The reader is referred to recent reviews for a more detailed summary [31,32]. Wet compounding was first described in a patent by Korbee et al. [33] in 1999 on an improved process for the preparation of PA nanocomposites. Liquid, preferably water, was injected into a twin screw extruder to improve the dispersion of nanoparticles (e.g., nanoclays) without the need for prior chemical modification of the nanoclay, which had previously been found necessary to achieve good exfoliation (i.e., dispersion). In later work, Fedullo et al. [34] described the mechanism for improved dispersion in such an approach with PA6-clay nanocomposites, reporting that water injected at high pressure and temperature had a two-fold effect: (1) lowering the viscosity and increasing the polarity of the PA6 and (2) diffusing into the galleries between nanoclay platelets and increasing their spacing. Both effects led to increased diffusion of the PA into the intergallery spaces, facilitating dispersion.

In a different approach, Hasegawa et al. [35] combined the unmodified nanoclay and water into a slurry and then pumped it into the extruder. The authors stated that the water evaporated from fine slurry drops in contact with molten PA, leaving behind exfoliated clay layers fixed onto the PA6. Vigorous blending and rapid water removal were thought to be keys to achieving good dispersion. Properties were similar to those obtained with organoclays, but the approach used a large amount of water to reduce the viscosity of the nanoclay slurry, which contained only 2% nanoclay. The same research team later injected water into a molten mixture of polypropylene (PP), nanoclay, quaternary ammonium salt, and maleated polypropylene (MAPP) [36]. The concept was to perform an in situ organomodification of the nanoclay and use the MAPP as a dispersant. Similar performance was found as with conventionally processed PP/organoclay nanocomposites where the organomodification was conducted prior to extrusion.

In subsequent work, many researchers have continued to try and refine wet extrusion compounding by reducing the amount of water used or expanding its use to other types of nanoparticles and polymers, for example [31]. While some batch mixers have been used in wet compounding investigations, the majority of research has been conducted using twin screw extruders [31]. Shahabadi et al., [37] identified three general categories of wet extrusion compounding: (1) slurry injection, (2) solution injection, and (3) water injection based on whether the composition of the liquid pumped into the extruder was a nanoparticle slurry, a surfactant or modifier solution, or water only (Table 1). Usually, the slurry, solution, or water is pumped into a high-pressure zone created through appropriate screw design, often with the aid of sealing rings to prevent the water from flashing off until after the downstream sealing ring [36]. Those materials not mixed with the liquid as a solution or slurry are metered into the main feed throat. The modifiers in the solution injection approach need to be water-soluble or at least dispersible. The need for such modifiers and compatibilizers depends on the polymer and nanoparticle used. Often quaternary ammonium compounds are used as modifiers with nanoclays, which can ion exchange with sodium ions on the nanoclay surface. Compatibilizers such as maleated polyolefins are common with polypropylene and polyethylene, for example.

The three approaches have different advantages and disadvantages as described in Table 1 and certain approaches may be more appropriate for a specific polymer or nanoparticle. For example, since the water injection approach is the least likely to modify nanoclay, it is more appropriate for polymers where such modifications may not be necessary (e.g., PA) or have been performed prior to extrusion. With respect to CNs, the solution and water injection approaches would require drying of the CNs prior to extrusion, likely involving costly freeze- or spray-drying. The resulting low bulk density material would be difficult to feed and meter into an extruder. Also, complete dispersion at the nano-scale is challenging when using dried CNs, and anything short of this may negate the advantage of using a nano-scale material in the first place. The slurry injection approach may be appealing for CNs since they are already produced as aqueous suspensions and since this would eliminate the need to first dry the CNs. However, such an approach for CNs may suffer from the same disadvantages as when nanoclays are used (i.e., high water content and low extrusion rates). Consequently, it will be important to understand how high of a CN concentration is possible that still can be easily fed and metered into an extruder and then dispersed in a polymer.

Table 1 certainly does not encompass all possibilities, and cellulose-based micro/nanomaterials may offer unique opportunities that are not necessarily appropriate or relevant for nanoclays. For example, microfibrillation of cellulose pulps has been shown to be possible in an extruder and will be discussed later. There are also unique challenges for micro- or nanocellulose materials compared to other materials of similar scale. For instance, their low thermal stability makes blending them with high melt temperature thermoplastics such as PAs challenging, even though PAs are more compatible with CNs than polyolefins, for example. Clearly, wet compounding of CN composites is not necessarily straightforward. However, progress has been made and the technology appears promising. Next, we discuss the specifics of approaches that have been explored for wet compounding of CNs.

### 2.3. Wet Extrusion Compounding of CNs

Twin screw extrusion is the most commonly used method for the wet compounding of cellulose nanocomposites [6,9,12,38,39,40,41,42,43,44,45,46,47,48,49,50,51,52,53,54,55,56,57]. Extruders are widely available and typically have multiple zones that can individually be tailored for mixing intensity, temperature, venting, etc., making them very adaptable. Multiple approaches have been used to produce cellulose nanocomposites by wet extrusion compounding.

#### 2.3.1. Compounding of CN Suspensions

In some cases, CNs have been introduced into extruders as aqueous suspensions (Table 2), which is analogous to the slurry injection approach for nanoclays (Table 1). Favoring such an approach is perhaps not surprising given that other approaches such as solution or water injection would involve first drying the CN suspensions and then trying to rewet them in an extruder, which would be both inefficient and potentially problematic.

**Figure 3:**
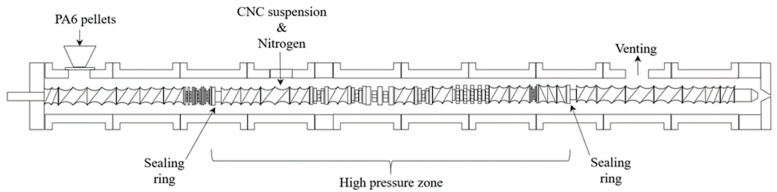
Wet twin screw extrusion compounding approach for PA6-CNC composites. Adapted from Peng, et al. [6], Polymer, Elsevier Ltd., 2016.

**Figure 4:**
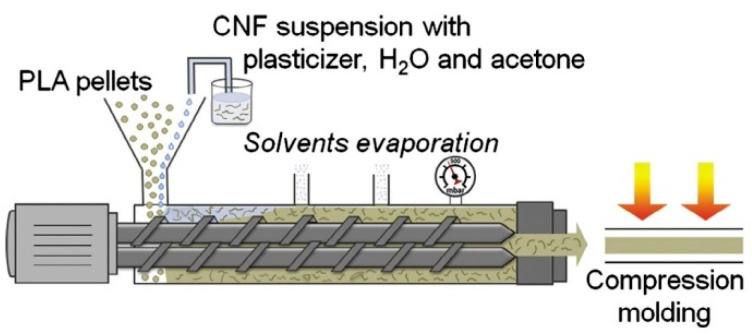
Liquid feeding process for plasticized PLA-CNF composites. Reproduced with permission from Herrera, et al. [54], Composites Science and Technology, Elsevier Ltd., 2015.

Peng et al. injected CNCs into polyamide 6 in the melt zone of a 32 mm twin-screw extruder to produce PA6-CNC nanocomposites with nanoscale dispersion (Figure 3) [6]. They pressurized the extruder to about 2 MPa with nitrogen to keep the water in a liquid state and to exploit the cryoscopic effect of PA6, allowing the reduction of the barrel temperature by 30 °C in the middle zones. They used sealing rings and screw design to maintain pressure, and water vapor was primarily vented downstream from this sealed zone. Adding well-dispersed CNCs reduced the cell size and increased the cell density and uniformity in foamed PA6 (Figure 1), improving mechanical properties and potentially allowing significant light-weighting of parts [6]. In their wet extrusion process, Stoeffler et al. injected 2.5% wt. aqueous suspensions of carboxylated CNCs into the melt zone and compounded them with PP or low density polyethylene LDPE [9]. They decreased the amount of aggregates by nearly a factor of 4 compared to melt blended composites when using polyolefins functionalized with polar groups, although there was still a large number of micrometer scale agglomerates visible [9]. With the inclusion of surfactants, they reported that wet compounding increased the tensile strength of their LDPE-CNC nanocomposites by 38%. Oksman et al. produced polylactic acid (PLA)-CNC [12,53,55] and PLA-CNF [54] by feeding CN suspensions into a twin screw extruder and then wet compounding with PLA. In their early work, a CNC suspension was produced by solvent swelling and sonicating microcrystalline cellulose (MCC), which was then concentrated and injected into the 4th of 11 zones of a twin screw extruder [12]. Solvent was then vented downstream. Detailed transmission electron micrographs demonstrated that submicron particle size could be achieved using this approach, if well-formulated [12]. In later work, rather than feeding the CNs downstream, they premixed a CNF suspension with plasticizer and solvent (to assist in dissolution of the plasticizer) and then fed the mixture into the main feed throat along with PLA pellets (Figure 4) [53,54]. Both atmospheric and vacuum venting was used. They found that wet compounding of CNs could improve the mechanical properties of plasticized PLA producing toughened composites but, since they used 20% plasticizer, the tensile moduli and strengths of the composites were lower than that of neat PLA.

**Table 2:** Cellulose nanocomposites produced by liquid or gel feeding of cellulose nanomaterials (CNs) via wet extrusion compounding.

Polymer	CN Type, Feed Conc., Final Conc.	Additive, Final Conc.	Compounding	Results	Reference
PLA	CNCs from MCC MFC by refining/cryocrushing, 4%, 5%	Polyethylene glycol (PEG), 5%	Polymer fed in 1st zone; CNC or MFC suspension fed at 4th zone by peristaltic pump; atmospheric venting at 7th and 8th zones and vacuum venting at 10th zone (of 11 zones); 100 rpm; 165–185 °C; 4 kg/h	No significant improvement in mechanical properties compared to PLA, which was attributed to non-uniform dispersion	Mathew et al. 2006 [58]
PLA Maleated PLA	CNCs from MCC ^1^ in DMAc ^2^ and LiCl 17%, 5%	Polyethylene glycol (PEG), 15%	Polymer fed in 1st zone; CNC suspension fed in 4th zone (of 11); 25 mm screw, 150 rpm, 5 kg/h, 170–185 °C; atmospheric and vacuum venting	Nanoscale dispersion with PEG; DMAc deteriorated PLA properties but CNCs improved DMAc-PLA controls	Oksman et al. 2006 [12]
PLA Polyvinyl alcohol (PVOH)	CNCs, ~3–4% ^3^ (in a 6:1 PVOH:CNC (aq) suspension), 5%	NaOH added to CNC-PVOH suspension, (0.25 mol/L of suspension)	CNC suspension fed by peristaltic pump downstream from main feed throat; 25 mm screw; 150 rpm, 4 kg/h, 170–200 °C; atmospheric and vacuum venting	PLA and PVOH were immiscible; CNCs were primarily found with PVOH; Increases in properties were attributed to reinforcing the PVOH phase	Bondeson and Oksman 2007 [55]
Cellulose Acetate Butyrate (CAB)	CNCs, 3.9%, 5%	Triethyl citrate (TEC), 15%	Polymer fed in main feed throat; CNC in water and ethanol with TEC fed downstream into melt; 25 mm screw; 150 rpm, 4.2 kg/h, 140–170 °C; atmospheric and vacuum venting	CNCs were dispersed in CAB; operating temperature of the nanocomposites increased from 100 to 140 °C; composites transparent	Bondeson et al. 2007 [59]
LDPE	Carboxylated CNCs neutralized with NaOH, N/A, ^4^ 2.5%	Cationic surfactant, N/A ^4^	CNC suspension fed into melt; sealing rings used; 180–200 °C, 5 kg/h, 34 mm screw	Slurry injection reduced CNC aggregates, especially for functionalized polymers; tensile strength of functionalized LDPE increased by 38% with surfactant	Stoeffler et al. 2013 [9]
PP					
LDPE w/5–10% polar groups					
PP w/0–1% polar groups					
PLA	CNF mechanically fibrillated from banana waste, 1.3%, 1%	Glycerol triacetate (GTA), 20%	CNF suspension (61% acetone/25% GTA/12% water) fed into main feed throat (with PLA pellets) using peristaltic pump; 3 kg/h total (removed 1.7–1.8 kg/h vapor), 300 rpm; 170–200 °C; atmospheric and vacuum venting	CNFs improved work of fracture, nearly doubling the effect of plasticizer	Herrera et al. 2015 [54]
PLA	Sulfated CNCs (sodium form), 2.6%, 1%	Triethyl citrate (TEC), 20%	CNC suspension (52% TEC, 22.7% water, 22.7% ethanol) fed into main feed throat (with PLA pellets) using peristaltic pump; 3 kg/h total, 300 rpm; 170–200 °C; atmospheric and vacuum venting	CNCs enhanced mechanical properties of plasticized PLA; mostly achieved nanoscale dispersion with some agglomerates; fast cooling yielded more transparent composites with higher elongation at break	Herrera et al. 2016 [53]
Polyamide 6 (PA6)	Sulfated CNCs (sodium form), 1.7–11%, 0.5–3.5%	-	CNC suspension injected into melt, which was sealed with sealing rings and reverse knead elements; water was maintained as liquid by pressuring with N_2_ to exploit cryoscopic effect of PA6, resulting in reducing temps in middle of extruder by 30 °C; vacuum venting at end	Nanoscale dispersion of CNCs, which acted as nucleation fillers for microcellular foaming; CNCs increased cell density and reduced cell size and improved mechanical properties of foamed composites.	Peng et al. 2016 [6]
Polyethylene (PE) Maleated PE	CNF mechanically fibrillated from Oil palm mesocarp fiber, 0.2%, 0.5-5%	-	Liquid fed at main feed throat with polymers; 80/160/160/160 °C; 50 rpm; compared to batch mixing; venting downstream	Improved tensile and flexural moduli and strength values compared to neat polymer and batch method; maximum properties around 3% CNF	Yasim-Anuar et al. 2020 [60]

^1^ Microcrystalline cellulose; ^2^ N, *N*-dimethyl acetamide; ^3^ Calculated based on other descriptions; ^4^ Not available.

One major disadvantage of these approaches involving the feeding of CN suspensions into a twin screw extruder is their low CN concentrations, which requires the venting of large amounts of water, limiting throughput and the final CN loading achievable in the nanocomposites. It is perhaps not surprising then that no composite in Table 2 contains more than 5% CN. Although small CN loadings may achieve the desired effect in the final composite, higher CN loadings (or higher throughputs) may require more concentrated CN suspensions. Such concentrating (a.k.a dewatering) of CN suspensions is an ongoing area of research [30]. However, one of the challenges of such an approach is that relatively small changes in concentration can lead to large changes in the viscosity, feeding characteristics, or ease of redispersibility depending on the type of CN. For example, even at a concentration of 2%, some CNF suspensions can become firm gels that are difficult to redisperse. Some researchers have also premixed CN suspensions with polymer powders, in part to improve the feeding characteristics (Table 3). Fine polymer powders are usually used to increase the surface area and reduce CN interaction, and also are more readily suspended in CN dispersions than pellets. For example, Hietala et al. [61] mixed CNF suspensions with potato starch prior to compounding them. Lo Re et al. [62] and Kaldéus et al. [63] partially dried mixtures of CNF suspensions, compatibilizer, and polycaprolactone (PCL) powder to 50% solids prior to extrusion compounding. Suzuki et al. [50] concentrated MFC to 20–25% prior to premixing with powdered PP and adhesion-promoting additives before compounding. Interestingly, Suzuki et al. found that this approach did not improve modulus and strength as much as the 2nd approach, discussed in the next section, in which the starting cellulose pulp was fibrillated in the extruder.

**Table 3:** Cellulose nanocomposites produced by pre-mixing CN suspensions with polymer powders followed by wet extrusion compounding.

Polymer	CN Type, Initial Conc., Final Conc.	Additive, Final Conc.	Mixing and Compounding	Results	Reference
Potato starch (powder)	Mechanical CNFs, 12%, 5–20%	D-sorbitol, 30% (of starch); stearic acid, 1% (of starch)	All components mixed in blender, giving “powdery state”; fed in main feed throat; 80–110 °C; 200 rpm; venting at zones 2 and 4 (out of 7)	CNF improved mechanical properties and favorably affected moisture uptake; transparency reduced but good even at 20%; some aggregation of CNFs	Hietala et al. 2013 [61]
PP (powder) MAPP	Mechanical CNFs 20–25%, 30%	Cationic polymer with primary amino group (CPPA), 6% or 9%	PP, MAPP, and CPPA mixed in food blender, then fed into extruder; 110–180 °C; 200 rpm; 200 g/h; they compared this pre-mixing method with extrusion fibrillation case	Significant improvement of tensile properties compared to PP but was not as good as the case in which they fibrillated the cellulose by extrusion	Suzuki et al. 2017 [50]
PCL (powder) PMMA (poly(methyl methacrylate)) nanoparticles	Enzymatic/mechanical CNFs, 1.6%, 10% or 20%	-	Polymers and CNF mixed and dried to 50% solids; used a microcompounder; 30 rpm for 5 min (feeding) and 100 rpm for 10 min; 120 °C	Wet feeding alone was better than dry feeding but using PMMA particles had bigger impact due to improved dispersion	Lo Re et al. 2018 [62]
PCL (powder)	CNF and MFC, 1.5% (CNF) and 2.4% or 10% (MFC), 3–5% (CNF) and 3-20% (MFC)	-	Compared coarse pulp, CNF and MFC; manually mix PCL and cellulose; used a microcompounder; 30 rpm for 5 min (feeding) and 100 rpm for 10 min; 120 °C; initial water contents 0–76%	Wet feeding gave improved mechanical properties for pulp; pulp performed as well as CNF; wet feeding preserves fiber length; low aspect ratio MFC did not improve mechanical properties much;	Lo Re and Sessini 2018 [64]
PCL (powder) Copolymer of 2-(dimethylamino)ethyl methacrylate and 2-hydroxy methacrylate	TEMPO ^1^—oxidized CNFs, 1%, ~10%	benzoyl peroxide (Luperox A75), 0.05–0.1%	Used a waterborne reactive nanoparticle compatibilizer to modify CNF first, then all components mixed and dried to 50% solids; used a microcompounder; 30 rpm for 5 min (feeding) and 100 rpm for 10 min; 140 °C	Their synthesized compatibilizer improved dispersion and properties; tensile and bending DMA showed significant increase in strength and stiffness	Kaldéus et al. 2019 [63]

^1^ (2,2,6,6-Tetramethylpiperidin-1-yl)oxyl.

#### 2.3.2. Compounding of Fibrillated Cellulose

In addition to aiding in dispersing CNs, water has also been used to facilitate the preparation of cellulosic materials of fine dimensions, included during wet extrusion compounding (Table 4). For example, Soulestin et al. 2007 [65] attempted to prepare cellulose nanofibers from MCC by injecting water into a high pressure section of a twin screw extruder while compounding with LDPE. The approach was somewhat analogous to the water injection process for clay nanocomposites (Table 1). A wide range of processing conditions were investigated and improvements in modulus were found, especially above 10% cellulose by weight, which was estimated to be the percolation threshold. The MCC was largely disaggregated in the process but cellulose dimensions appeared to be well above nano-scale [65]. 

However, Soulestin et al.’s approach was quite unique and the great majority of research on the topic has been on the fibrillation of coarse pulps (Table 4). A major advantage of such an approach is that the initial water content can be kept relatively low, reducing the amount of water that eventually needs to be vented. For example, Beaugrand et al. [66] investigated the effects of processing parameters on the fibrillation of water-plasticized hemp fibers while compounding them with PCL in a twin screw extruder. Fibrillation down to nanoscale was not necessarily the target, and the approach resulted in coarse fibrils, whose dimensions and effects on mechanical performance were shown to vary considerably with processing conditions. The investigation demonstrated that fibrillating, compounding, and drying are possible in a single pass through an extruder, even if the fibrillation did not result in nanoscale material. Much more recently, Lo Re et al. used a recirculating twin screw microcompounder to fibrillate and wet compound unmodified and acetylated cellulose fibers with PCL powder. The tensile properties of the composites produced were much higher than those produced by dry feeding, increasing the modulus and strength by 213% and 71%, for unmodified cellulose. Even higher improvements were found when acetylated fiber was used [42]. However, fibers were not of nanoscale dimensions and long processing times (20 min of recirculation) were used, partly due to the limited shearing capabilities of the microcompounder used.

**Figure 5:**
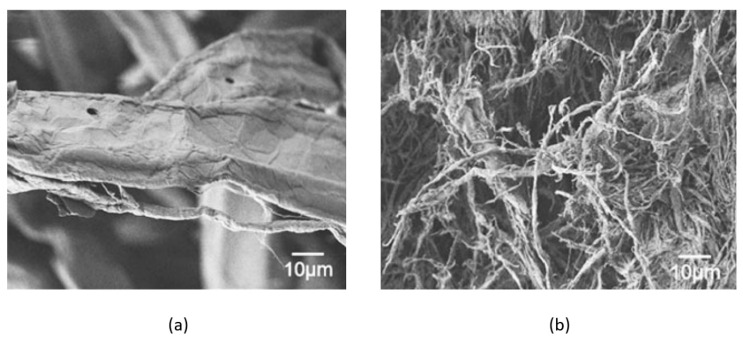
Starting pulp (**a**) and fibrillated cellulose from final composite after extraction of the polymer matrix (**b**). Reproduced with permission from Suzuki, et al. [38], Cellulose, Springer Nature B.V., 2013.

Beyond the references mentioned above, the great majority of research on extrusion fibrillation in wet compounding has been performed by a collaborative team of researchers in Japan. In a series of investigations, they explored producing cellulose microfibrils (or nanofibrils) from never-dried wood pulp by extrusion kneading with polymers, usually at sub-ambient barrel temperatures, followed by wet extrusion compounding at temperatures above the polymer melting point [38,39,40,41,45,50]. The process used the polymer as a co-grinding agent and partially fibrillated the cellulose resulting in fibril widths ranging from submicron to several micrometers (Figure 5). Low water contents facilitated higher outputs and production of composites with fibrillated cellulose loading levels as high as 60% were possible [38]. Modified pulps, such as by acetylation, were reported to be more easily fibrillated, resulting in fibrils with smaller diameters [41], potentially improving the efficiency. Polypropylene [38,39,40] and polyethylene [40] cellulose nanocomposites with improved mechanical and heat distortion properties were produced. In one study, they dissolved and removed the polymer matrix, and an electron micrograph of the remaining fibers showed a hierarchical structure with some sub-micron fibrils present [38]. They demonstrated a high level of cellulose fibrillation, and the resulting injection-molded composites had as much as double the tensile modulus of unfilled polymer samples. Heat distortion temperatures increased by more than 50 °C in some cases.

Some success has also been had drying mixtures of CNs (or MFC) and fine polymer particles (or emulsions) either by extrusion or by batch processes prior to extrusion compounding [43,44,45,47,48,51,67]. In such cases, the polymers and cellulose had high surface area and were well-mixed prior to the removal of water, so agglomeration was mitigated during drying. However, it is not clear how dry these mixtures were before compounding or whether residual moisture prevented hydrogen bonding and/or aided in compounding. For example, Yano et al. reported one case in which these mixtures contained 2.0% ethanol and 0.5% water after drying [45]. These studies suggest that it may be possible to produce quality cellulose nanocomposites by first wet mixing followed by removal of the vast majority of water (or other liquids) prior to compounding. Still, the challenge is to prevent cellulose from agglomerating as water is removed from the mixture and as it is compounded, and more work is clearly needed to determine the optimal strategies for mixing, compounding, and water removal.

#### 2.3.3. Water Effects on Molecular Weight and Crystal Structure

Adding water to polymers during extrusion can affect the crystal structure and molecular weight of polymers. Fortunately, the reported molecular weight degradation experienced by polymers during wet compounding by extrusion has so far been found to be minimal. For example, Peng et al. reported melt extrusion of PA6 with and without water led to reductions in molecular weight of 13% and 9.5%, respectively, a difference of less than 4% [6]. Lo Re et al. also reported minimal change to the molecular weight of PCL when wet compounded with cellulose [42,62], and in one case, the maximum reduction was less than 5% even when compounded up to 30 min or with up to 41% initial water content [42]. Peng et al. [6] also reported that water led to the nucleation of alpha phase crystal structure of PA6, which favorably affected the mechanical properties of PA6, even in the absence of CNCs. The effect of water-assisted extrusion on biopolymer molecular weight is not yet clear. Oksman et al. reported multiple efforts to produce PLA-CN nanocomposites by wet extrusion compounding, but they did not report the molecular weight of PLA [12,53,54,55]. Furthermore, processing aids or plasticizers had negative effects on the PLA making it difficult to determine if the presence of water led to significant hydrolytic degradation of PLA. Consequently, more work is needed to evaluate the efficacy of wet extrusion compounding using PLA and other biopolymers. Although the number of studies is quite limited, changes in polymer morphology during water-based extrusion have so far shown to be minimal and do not appear to adversely affect the properties of polymers.

#### 2.3.4. Water Effects on Additives

Wet compounding by extrusion does not appear to interfere with the use of traditional additives (coupling agents, etc.,) and presents opportunities to explore aqueous-based additives and modification strategies. Suzuki et al. demonstrated improved mechanical properties of PP-cellulose nanocomposites with 30% cellulose up to about 4–5% MAPP addition, showing little indication that water had severely hydrolyzed MAPP or otherwise interfered with its effectiveness as a coupling agent [38]. Suzuki et al. also later reported that the use of water-based cationic polymers further improved the properties of PP and microfibrillated cellulose composites [39,40]. These water-soluble polymers were simply mixed in with the wet cellulose and polymer powder fed into the extruder. These cationic polymers were effective in improving nanocomposite properties without a separate modification step. Surfactant-modified cellulose nanomaterials have also been effectively introduced into extruders as aqueous suspensions [9]. Researchers also described using other water-soluble solvents or liquids, such as alcohol or acetone, as part of the wet compounding process (e.g., to swell polymers and to improve dispersion of modified CNs) [45,46,49,53,54]. The ability to use traditional polymer processing additives, as well as water-based cellulose modification strategies, may prove advantageous in the wet compounding of cellulose nanocomposites.

In summary, approaches to wet extrusion compounding can divided into two main categories: (1) feeding and compounding of previously prepared CN suspensions or (2) combined/sequential fibrillation and wet compounding of pulps. Both approaches have their advantages and disadvantages and there are many variations within each category. The nanocomposites produced generally show improved dispersion and properties compared to the dry melt-blending of nanocomposites, often resulting in superior performance to neat polymers. Investigations so far have not shown major problems with hydrolysis of polymers or additives in optimized processes. However, technical and practical challenges remain. For example, dispersion of CN suspensions in polymers is often incomplete and the large amount of water needing to be vented can negatively impact the throughputs. More concentrated CN suspensions, more efficient water removal, or new approaches may be necessary. Pulp fibrillation approaches have the advantage of lower initial water content and potentially faster throughputs if the fibrillation can be performed efficiently. Optimization of the fibrillation of pulps, whether performed prior to or in the same step as wet compounding is on-going.

### 2.4. Wet Batch Compounding of CNs

Batch mixers have also been used in wet compounding of CNs with various polymers and recent research is summarized in Table 5. Because of the different nature of the equipment, approaches are necessarily quite different than with a continuous mixer such as a twin screw extruder. In extrusion, materials move through different zones that have been configured to perform different functions such as melting, compounding, or devolatilization. As previously mentioned, a particular modification for some twin screw wet compounding approaches is the configuring of a high pressure zone so that water remains liquid at the melt temperature of the polymer matrix used [31]. With batch mixers, processing parameters such as rotor speed can be varied over time but the basic configuration of the mixer remains constant. Despite this limitation, batch mixers have proven useful in wet compounding of cellulose nanocomposites.

For example, when compounding CNCs in a roller blade mixer, Sapkota et al. [7] made the simple observation that the presence of even small amounts of water during the compounding of lyophilized CNC and a fine LDPE powder can greatly improve the dispersion in cellulose nanocomposites. About 10% water by weight appeared optimal for avoiding large-scale aggregation of CNCs. Although the level of dispersion was not evaluated, the compression-molded thin films did show good transparency and improved the mechanical performance over neat LDPE films and composite films prepared with dry, lyophilized CNCs. Interestingly, the authors also note that compounding times below 10 min led to some voids in the thin films as a result of residual moisture in wet compounded films despite a compounding temperature of 170 °C. In another example, Suzuki et al. used a twin rotary roller mixer to wet compound PP, MAPP, and cellulose that was previously fibrillated by extrusion [38]. When the composites were subsequently compression molded, major improvements in mechanical performance were found when up to 50% of the microfibrillated cellulose was added. The microfibrillated cellulose was also wet extrusion compounded but the compound was then injection molded rather (than compression molded), making direct comparisons between the two approaches difficult. However, the purpose of doing so was to verify that a continuous wet compounding process could also be used rather than to compare the two approaches. 

Recently, we have investigated using a thermokinetic mixer for wet compounding of cellulose nanocomposites [8,10,11,68,69]. Though somewhat rare, thermokinetic mixers are fully fluxing, ultrahigh-intensity batch mixers. Materials are heated through frictional forces and once fluxed, shear rates of about 10^4^ s^-1^ can be reached [70], which is at least an order of magnitude higher than that typically found for twin screw compounding [70]. Gopakumar et al. [70] proposed a useful distinction between the induction and mixing times, defined as the time required to reach the onset of polymer melting (induction time) and the time in which the polymer melts and is mixed with other constituents (mixing time).

Because of their high shear rates and short mixing times, thermokinetic mixers have been used to compound plastics with nanoparticles such as organoclays [70,71,72] or graphite [73] or thermally sensitive fillers and reinforcements such wood [74,75,76] or other natural fibers [77,78,79]. Therefore, it is perhaps not surprising that thermokinetic mixers have been used to compound plastics with CN suspensions as well. Their short compounding times can minimize not only the thermal degradation of the CNs but the potential hydrolysis of susceptible additives or matrices, such as bio-based polymers (e.g., PLA). For example, Sabo et al. [11] found no reduction in PLA molecular weight in cast films when CNC was added if blends were wet compounded quickly (total batch times (induction + mixing) of less than 2 min) prior to film extrusion. This was true even with a water:PLA weight ratio of about 1:1 during wet compounding.

**Figure 6:**
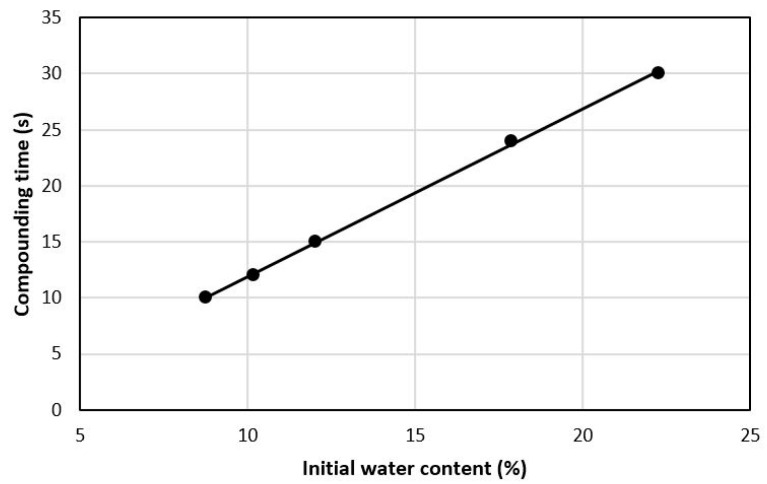
Effect of water content on batch time for the thermokinetic mixing of PP-CNC composites [80].

Not surprisingly, shorter compounding times can be achieved with even less water, which effectively shortens the induction time. For example, Figure 6 shows the effect of overall water content on the total batch time of polypropylene (PP) with 5% CNCs by weight at water contents of up to 22% of the total weight [80]. The linear relationship with water content is quite clear as are the very short compounding times when little water is present. Such short compounding times are well below those used with other batch compounders or the residence time of twin screw extruders. The apparent efficiency of water removal by thermokinetic mixers suggest that they may offer an opportunity to both flash dry and compound cellulose nanomaterials with polymers without the need for a separate drying process. This has already proven useful as a matter of convenience in a laboratory setting. However, determination of the minimum amount of water necessary for good dispersion as well as the efficiency and relative costs of drying in a thermokinetic mixer versus other drying methods still need to be evaluated to support broader use.

**Figure 7:**
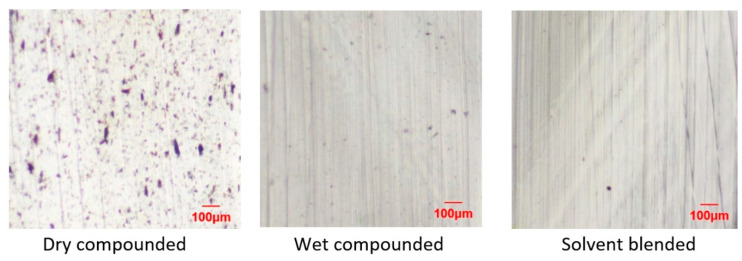
Microscope images of sections of PA6-CNC composites prepared by dry compounding of freeze-dried CNCs, wet compounding of CNC dispersion, and solvent blending in formic acid [8]. All composites contain 5% CNC by weight.

**Figure 8:**
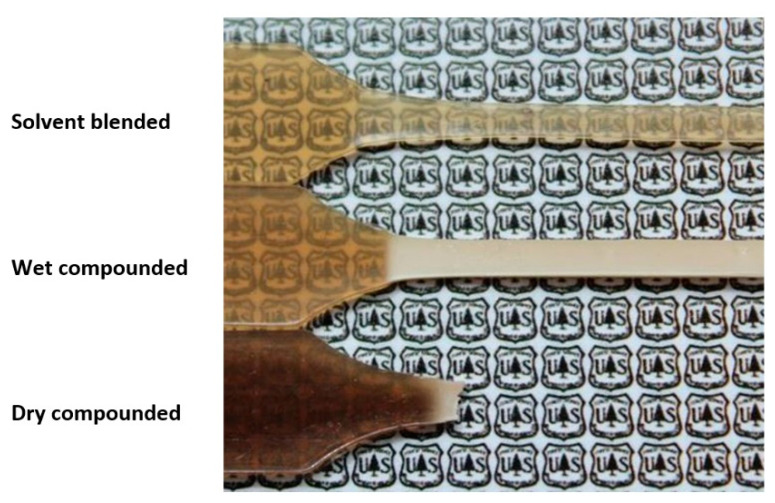
Injection molded PA6 specimens containing 5% CNCs after tensile testing [8].

Despite the inability to create a high-pressure environment as in some of the wet extrusion compounding approaches, the presence of water when compounding in a thermokinetic mixer has also led to major improvements in dispersion. Clemons [8] showed much improved dispersion when wet compounding CNCs in PA6 in a thermokinetic mixer compared with dry compounding of freeze-dried CNCs (Figure 7). However, dispersion was not as good as with a solvent blending approach suggesting that, while wet compounding shows promise and can avoid the use of solvents, there is still room for improvement. The differences in dispersion of CNs in injection molded composites made from the compounds was very clearly seen in the mechanical performance of the composites, especially the elongational behavior (Figure 8). Interestingly, discoloration due to the limited thermal stability of CNCs was not any worse than with the solvent-blended approach, nearly all of which occurred during injection molding of the dry compound.

While the presence of water during compounding has clearly been shown to improve CNC dispersion, the precise mechanism of the improved dispersion needs further exploration. Interestingly, despite discharging the composite melt well above the boiling point of water, residual moisture is readily apparent as is evident by the steaming of the melt after discharge and the presence of voids if immediately compression molded. How much of this residual moisture is due to: (1) the very rapid temperature increase after much of water is removed versus the finite time necessary for complete water removal, (2) temperature inhomogeneity (e.g., localized temperature differences between material under blades vs. bulk) or (3) other factors, requires further investigation. This residual moisture likely aids in dispersing the CNs, in part, by disrupting hydrogen bonding. Also, the presence of moisture in the melt could potentially act in a similar manner as supercritical or even subcritical fluids (e.g., nitrogen or carbon dioxide), which have been shown to facilitate the dispersion of nanoparticles during melt compounding [81,82]. Moisture volatilization and steam expansion in the polymer melt could similarly create extensional flows, imparting greater forces on polymer agglomerates than in shear, which predominates in conventional processing [82]. A greater understanding and optimization of these mechanisms should be undertaken to further improve the CN dispersion in wet compounding.

**Figure 9:**
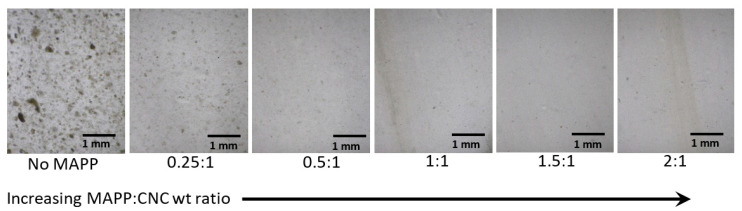
Thin films showing the effect of MAPP on the dispersion of CTAB-treated CNCs in PP. Materials were wet compounded in a thermokinetic mixer [68].

**Figure 10:**
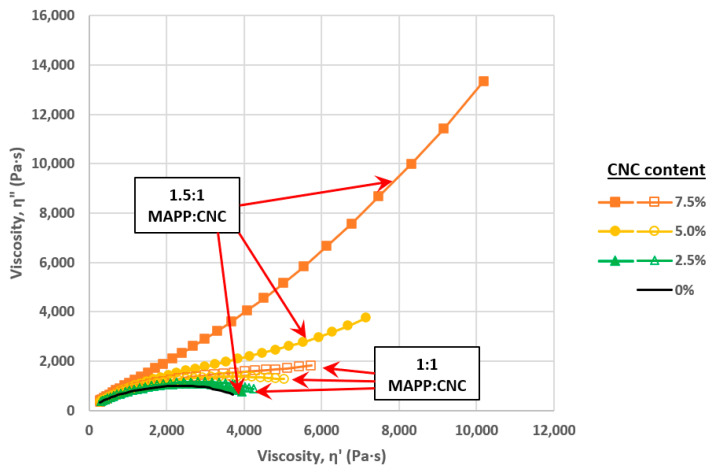
Effect of CNC content in small amplitude oscillatory shear tests on thin films with 1:1 or 1.5:1 MAPP:CNC wt. ratio. Tests were conducted at 190 °C and a 1% strain [68].

Not surprisingly, batch compounding of CNs with hydrophobic polymers such as polyolefins is problematic and often involves treatments, dispersing agents, or a combination of both. For example, Clemons and Reiner [68] explored additives and treatments similar to those used in the wet compounding of nanoclays in polyolefins [36,37]. An aqueous quaternary ammonium treatment was first used to render CNCs more hydrophobic and to help reduce the potential for hydrogen bonding. Also, a maleated polypropylene (MAPP) was added during wet compounding in a thermokinetic mixer to facilitate CNC dispersion in PP (Figure 9). Characteristic changes in the Cole-Cole plot (Figure 10) and other rheological plots (not shown) suggests increased network formation at higher MAPP content [83]. This likely results from greater CNC-polymer interaction, greater CNC-CNC interaction, or both [84]. However, it seems likely that improved dispersion also plays at least a partial role given the trend in Figure 9. The authors also demonstrated that dispersion increased with smaller batch size, likely due to an increase in specific mechanical energy (i.e., mechanical energy input/kg of compounded material).

In summary, recent research in batch wet compounding has demonstrated promising improvements in the dispersion of CNs in polymers. Also, process optimization and short mixing times have mitigated negative effects such as hydrolysis of susceptible polymers and additives. However, further improvements are still necessary to achieve optimal dispersion, improve efficiency, and optimize treatments and formulation. A deeper understanding of process dynamics and the mechanistic details of dispersing CNs in polymers during wet compounding would help inform optimization efforts.

## 3. Summary and Outlook

Production of CN-thermoplastic compounds involves: (1) preparing CNs, (2) removing water from the CN dispersion, (3) perhaps modifying surface chemistry to improve compatibilization with the polymer, and (4) dispersing the CNs in polymers. In early investigations, each of these steps was often performed separately. This proved useful in exploring the effects and the potential of material/process variables and approaches within each step but were not always efficient, environmentally friendly, or practical. Large-scale commercialization demands more commercially relevant, integrated approaches that improve efficiencies and reduce overall costs. For example, rather than simply being a problem to overcome after CN preparation, water removal could be managed more holistically over the entire composite preparation process, exploiting the characteristics of CNs to provide opportunities to aid in dispersion, fibrillation, and surface treatments.

Compounding of cellulose suspensions that are already of nanoscale dimensions have the potential to be truly nano-scale biocomposites, with their associated benefits (e.g., transparency, very high interfacial area) if they can be dispersed well and efficiently. In terms of overall water management, wet compounding approaches that judiciously retain water from CN suspensions can lead to major improvements in the dispersion of CNs in polymers. While wet compounding approaches have shown promise, the technology is still in its infancy and demonstration of very good dispersion with commercially relevant processes and high throughputs has been elusive. Without (nearly) complete dispersion, it may be difficult to justify the added cost of its use over less-expensive micro-scale cellulose materials (e.g., microcrystalline cellulose), since that is what CN agglomerates essentially are. Also, the high water content of CN suspensions often requires major water removal, limiting throughput, reducing efficiency, and increasing costs. Consequently, a greater understanding of both interactions among CN, water, polymer, and additive as well as details of the concentrating, drying, and dispersion mechanisms are necessary to further improve dispersion and process efficiency. This understanding would support efforts to optimize CN treatments/modifiers/formulations, identify optimal water content of CN suspensions that can be easily fed, metered, and dispersed in a plastic, develop more efficient water removal, and maximize throughput.

In addition to aiding in dispersing CNs, water has also been used to facilitate fibrillation of cellulose pulps, especially during extrusion compounding. One attraction of such an approach is that the starting pulps (rather than CN suspensions) can be concentrated more easily, resulting in less water removal and potentially higher throughput. They are also initially less viscous and can be easier to feed and meter. While some have tried to fibrillate, dry, and compound in a single step, these have not necessarily led to complete fibrillation or have not proven very efficient yet. Multiple passes or long circulation times are often used to compensate. Consequently, such approaches have often been combined with other grinding/fibrillation methods as well as chemical pretreatments, or polymers as co-grinding agents, for example, to improve efficiency or reduce dimensions prior to or during compounding. Even so, the result may ultimately not be fibrillation down to nano-scale widths nor should it necessarily be the ultimate goal. Identification of an optimal level of fibrillation that provides best balance of property improvements over the initial pulp without increasing cost too much may be more appropriate.

As technologies of wet compounding of CNs continue to be developed, they need to be demonstrated on a large scale, and the efficiencies and economics of various processes such as drying or fibrillating during the compounding step need to be assessed and compared to alternatives. The resulting composites need to be benchmarked against those containing other forms of cellulose, other biobased materials, or conventional alternatives and their value propositions demonstrated.

Finally, the perception that water has no value in polymer processing and must always be removed to avoid its negative effects should be revisited. Potential issues relating to hydrolysis of susceptible polymers or additives (e.g., PAs, biopolymers, MAPP) have not yet been found to be severe or have been mitigated through process optimization. While further investigation is definitely warranted, new opportunities for water use should also be considered, and a more sophisticated understanding of water’s role should be developed. This will become increasingly important as bio-based materials in thermoplastic composites continue to grow.

## Figures and Tables

**Table 1:** A description of the three different types of water-assisted melt intercalation and their advantages and disadvantages. Adapted from Shahabadi, et al. [37], Express Polymer Letters, Budapest University of Technology and Economics, 2012.

	Slurry Injection (SI)	Solution Injection (SoI)	Water Injection (WI)
Main feed:	Polymer Nanoclay (NC) modifier Compatibilizer	Polymer Compatibilizer NC	Polymer NC modifier Compatibilizer NC
Downstream feed:	NC slurry	NC modifier solution	Water
Advantages:	Best NC dispersion Many polymers and modifiers possible	Best NC dispersion Better NC dispersion than in WI Less water than in SI High output/NC content possible	Least water needed High output/NC content possible
Disadvantages:	Much water needed Slurry limited to ≤5% NC Low output	More water than in WI Only water-soluble modifiers	No sol’n/slurry preparation NC modification less likely Best when NC modification not necessary

**Table 4:** Cellulose composites produced by extrusion fibrillation and wet extrusion compounding.

Polymer	Cellulose: Feed Type, Feed Concentration, Composite Conc.	Additive, Final Conc.	Fibrillation and Compounding	Results	Reference
PP (powder) MAPP	Never-dried bleached and unbleached Kraft pulp, 20–25% solids, Composites with up to 60% cellulose	-	Fibrillated and compounded in separate extrusion steps with 15 mm screw, L/D of 45; fibrillation extrusion described as kneading: 0 °C, 400 rpm, 400 g/h; compounding at 110–180 °C, 200 rpm, 300 g/h, venting at middle and end zone	After matrix dissolved, hierarchical fiber structure with diameters from sub-micron to 10s µm; tensile, flexural, and impact properties improved; tensile modulus doubled and strength increased 1.5 times; heat distortion increased by 53 °C; unbleached pulp resulted in higher tensile strength	Suzuki et al. 2013 [38]
PP (powder) MAPP	Never-dried bleached and unbleached Kraft pulp, 20–25% solids, Composites with 30–40% cellulose	Cationic polymer with primary amino group (CPPA), 3%	Fibrillated and compounded in separate extrusion steps with 15 mm screw, L/D of 45; fibrillation extrusion described as kneading: 0 °C, 400 rpm, 400 g/h; compounding at 110–180 °C, 200 rpm, 200 g/h, venting at middle and end zone	Fibers shown after fibrillation appear hierarchical with micron and sub-micron fibers; composites had improved tensile properties compared to PP; CPPA further improved tensile properties	Suzuki et al. 2014 [39]
High density polyethylene (HDPE) PP MAPP	Never-dried bleached pulp, 20–25% solids, Composites with 20% cellulose	Cationic polymer with primary amino group (CPPA), 4%	Fibrillation and compounding done in separate extrusion steps with 15 mm screw, L/D of 45; fibrillation extrusion described as kneading: 0 °C, 400 rpm, 400 g/h; compounding at 110–180 °C, 200 rpm, 200 g/h, venting at middle and end zone	After matrix dissolved, hierarchical fiber structure with diameters from sub-micron to ~10 µm; greater improvements of mechanical properties with HDPE than PP; fibrillation better in HDPE than PP; MAPP and CPPA improve fiber-polymer interaction; improved heat deflection and thermal expansion	Suzuki et al. 2016 [40]
PP (powder) MAPP	Never-dried bleached pulp, 20–25% solids, Composites with 30% cellulose	Cationic polymer with primary amino group (CPPA), 6% or 9%	Same as Suzuki et al. 2014; [40] compared to pre-mixing wet CNFs with PP, MAPP, and CPPA in a blender, followed by wet compounding at 110–180 °C, 200 rpm, 200 g/h	Twin screw fibrillation combined with subsequent wet compounding had higher tensile properties than first producing CNFs followed by wet compounding	Suzuki et al. 2017 [50]
Various examples, including HDPE, PP, and PLA	Various pulps, including alkenyl succinic anhydride (ASA)-modified never-dried bleached pulps	Various amounts of CaCO_3_, nucleating agents, antioxidants	This patent describes various combinations of water and solvent-based modification, fibrillation, and compounding; they suggest water content during compounding be less than 20%	Various examples showed modified cellulose resulted in composites with improved properties; they claim nanoscale fibrillation	Yano et al. 2016 [45]
LDPE	Microcrystalline cellulose (MCC), 92% solids, Composites with 5–30%	-	Attempted to use high-shear wet extrusion compounding to liberate MCC into CNCs; they mixed the MCC powder with polymer in the melt zone and injected water downstream; 100–1200 rpm, 150 °C	Using water and especially polymer powders instead of pellets improved dispersion; water improved discoloration from cellulose in composites; tensile stiffness improved, especially above 10% cellulose and elongation decreased	Soulestin et al. 2007 [65]
PCL	Hemp fibers, 77.5–90% solids, Composites with 20% fibers	-	High-shear extrusion was used to fibrillate and compound hemp fibers with PCL; fibers added either with PCL or downstream; 25 mm screw, L/D of 36; 100 °C or 140 °C; 100–400 rpm; 0.85 kg/h or 1.5 kg/h	Severe fibrillation occurred but not to nanoscale; tensile modulus and strength improved by factor of 2 or 3; Factors leading to higher fiber aspect ratio (e.g., higher moisture, 100 °C instead of 140 °C) generally gave higher tensile properties	Beaugrand and Berzin 2013 [66]
PCL (powder)	Bleached softwood Kraft pulp, acetylated and not 22% solids, Composites with 5–20% cellulose	-	PCL powder and pulp slurry were pre-mixed and fed into a twin screw microcompounder at 120 °C and 30 rpm for 5 min; following feeding, 100 rpm for ~15 min until water presumed evaporated	Cellulose was highly fibrillated but not to nanoscale; acetylated fibers performed best with tensile modulus and strength increased by (860% and 150%, respectively)	Lo Re et al. 2018 [42]

**Table 5:** Cellulose nanocomposites produced by via wet batch compounding.

Polymer	CN	Additives	Compounding	Results	Reference
Fine powder of low density polyethylene (LDPE)	0–15% CNCs/MCC	None	LDPE premixed with CNCs/MCC dispersion; dried to 8–10% moisture; wet compounded using roller blade mixer and then compression molded	Presence of water and premixing improved dispersion (visual assessment); increases in modulus and strength, reduction in strain to failure	Sapkota et al. 2017 [7]
PE	0.5–5% oil palm CNF	3% Maleated PE	0.2% CNF dispersion wet compounded in internal mixer then compression molded. Compared with compression molded composites made from aligned strands from wet extrusion compounding.	Increases in strength and modulus over unfilled for all composites. Wet extrusion compounding yielded better dispersion than wet internal mixer compounding. Alignment of extruded filament led to alignment in composites and better strengths and moduli.	Yasim-Anuar et al. 2020 [60]
PP powder	Up to 60% MFC from never-dried bleached/unbleached Kraft pulp	Up to 6% MAPP	20–25% solids pulp mixed with PP and MAPP then fibrillated in an extruder. Mixture was then wet compounded in a twin rotary roller mixer, ground, and compression molded.	Microfibrillated cellulose (MFC) did not appear to aggregate during wet compounding. Strengths and moduli increased over unfilled, failure strains decreased. Approach was successfully transferred to wet extrusion compounding process.	Suzuki et al. 2013 [38]
PLA powder	1% CNCs	DTAC ^1^ (1:1 molar ratio with sulfates) 0 or 10% PVAc ^2^	Polymer and CNC suspensions were pre-mixed prior to compounding in thermokinetic mixer at either 3700 or 6000 rpms. Discharge temperatures of 155 °C and 175 °C. Compounds were ground and films were extruded.	DTAC and PVAc improved dispersion and transparency. Samples compounded at high speed (low residence time) did not have significant molecular weight degradation	Sabo et al. 2020 [11]
Polyamide 6 (PA6)	5% CNCs	None	Three compounding methods compared: Dry compounding of freeze-dried CNCs with PA6 in a thermokinetic mixer. Wet compounding of CNC dispersion with PA6 in a thermokinetic mixer. Solvent-blending of freeze-dried CNCs and PA6. All compounds were then injection molded.	CNC dispersion in wet compounding much better than dry but not as good as solvent blending. Dispersion particularly influenced elongational properties of composites. Thermal degradation of CNCs in wet compounded and solvent blended composites similar. Dry compounded much worse.	Clemons 2017 [8]
PP powder	Up to 7.5% treated CNCs	MAPP:CNC wt ratio of 1:1 or 1.5:1	Wet compounding of treated CNC dispersion (10% solids), PP, and MAPP followed by compression molding.	CNC dispersion well below micron but not perfect. Rheology showed good network formation above percolation (~4.5%) at high MAPP level.	Clemons and Reiner 2020 [68]
PLA powder	1% CNCs, with and without lignin	None	CNC suspension and PLA powder pre-mixed prior to compounding in thermokinetic mixer at 5500 rpm. Evaluated discharge setpoints of ~100 °C and 180 °C. Ground and extruded films.	Wet compounding resulted in films with improved mechanical and water vapor barrier properties compared to melt mixing freeze-dried CNCs. Lignin-containing CNCs did not perform as well	Sabo et al. 2019 [10]
PLA powder	2% wood CNFs, with and without lactic acid esterification	None	PLA and CNF suspensions were pre-mixed prior to compounding in thermokinetic mixer. Discharge temperatures of 140 °C. Compounds ground and injection molded.	Esterification did not dramatically change CNFs but resulted in more transparent composites than CNFs without lactic acid grafted.	Lafia-Araga 2018 [69]

^1^ Dodecyl trimethyl ammonium chloride; ^2^ polyvinyl acetate.

## Data Availability

Not applicable.
